# Kinomic profile in patient-derived glioma cells during hypoxia reveals c-MET-PI3K dependency for adaptation

**DOI:** 10.7150/thno.54741

**Published:** 2021-03-05

**Authors:** Hong Sheng Cheng, Charlie Marvalim, Pengcheng Zhu, Cheng Lui Daniel Law, Zhi Yan Jeremy Low, Yuk Kien Chong, Beng Ti Ang, Carol Tang, Nguan Soon Tan

**Affiliations:** 1 School of Biological Sciences, Nanyang Technological University Singapore, Singapore 637551, Singapore.; 2 Lee Kong Chian School of Medicine, Nanyang Technological University Singapore, Singapore 308232, Singapore.; 3 Neuro-Oncology Research Laboratory, Department of Research, National Neuroscience Institute, Singapore 308433, Singapore.; 4 Department of Neurosurgery, National Neuroscience Institute, Singapore 308433, Singapore.; 5 Duke-National University of Singapore Medical School, Singapore 169857, Singapore.; 6 Department of Physiology, Yong Loo Lin School of Medicine, National University of Singapore, Singapore 117593, Singapore.

**Keywords:** glioblastoma, HIF-1α, oxidative stress, PI3K, tumor microenvironment

## Abstract

Hypoxic microenvironment is a hallmark of solid tumors, especially glioblastoma. The strong reliance of glioma-propagating cells (GPCs) on hypoxia-induced survival advantages is potentially exploitable for drug development.

**Methods:** To identify key signaling pathways for hypoxia adaptation by patient-derived GPCs, we performed a kinase inhibitor profiling by screening 188 small molecule inhibitors against 130 different kinases in normoxia and hypoxia. Potential kinase candidates were prioritized for *in vitro* and *in vivo* investigations using a ranking algorithm that integrated information from the kinome connectivity network and estimated patients' survival based on expression status.

**Results:** Hypoxic drug screen highlighted extensive modifications of kinomic landscape and a crucial functionality of c-MET-PI3K. c-MET inhibitors diminished phosphorylation of c-MET and PI3K in GPCs subjected to hypoxia, suggesting its role in the hypoxic adaptation of GPCs. Mechanistically, the inhibition of c-MET and PI3K impaired antioxidant defense, leading to oxidative catastrophe and apoptosis. Repurposed c-MET inhibitors PF04217903 and tivantinib exhibited hypoxic-dependent drug synergism with temozolomide, resulting in reduced tumor load and growth of GPC xenografts. Detailed analysis of bulk and single-cell glioblastoma transcriptomes associates the cellular subpopulation over-expressing c-MET with inflamed, hypoxic, metastatic, and stem-like phenotypes.

**Conclusions:** Thus, our “bench to bedside (the use of patient-derived GPCs and xenografts for basic research) and back (validation with independent glioblastoma transcriptome databases)” analysis unravels the novel therapeutic indications of c-MET and PI3K/Akt inhibitors for the treatment of glioblastoma, and potentially other cancers, in the hypoxic tumor microenvironment.

## Introduction

Hypoxia, a phenomenon depicted by low oxygen bioavailability, is a hallmark of the tumor microenvironment (TME). In solid tumors, hypoxic regions often arise because of rapid tumor cell proliferation, which outgrows the oxygen perfusion and nutrient supply from normal vasculature 1. Upon sensing low intracellular oxygen concentration, hypoxia-inducible factors (HIFs) are activated to regulate the expression of genes with hypoxia-responsive elements, i.e., HIF gene signature. The resultant phenotypic changes include pro-angiogenesis, a transition from oxidative to glycolytic metabolism, and altered drug response 1. Hypoxia significantly enhanced cell motility and the expression of mesenchymal biomarkers like Twist, N-cadherin, and vimentin in various cancers 2, 3. Moreover, hypoxia-induced autophagy, which is closely linked to therapy resistance, removes and recycles damaged cellular compartments to prolong their survival 4. Such a tumor protective effect of hypoxia has been widely reported against radiotherapy and chemotherapy. Collectively, hypoxic TME is undeniably a predominant player in the plasticity, invasiveness, and drug resistance of tumor cells, rendering it an exploitable feature for cancer drug development.

Glioblastoma (GBM) is a devastating form of brain cancer with a dismal median survival duration, a high level of resistance to current therapy, and common recurrence after treatment. The median survival rate of GBM is 15 months after diagnosis, with fewer than 7% of the patients surviving longer than five years 5. The current standard therapy for GBM includes maximum debulking surgery, radiation, and treatment with the monofunctional alkylating agent temozolomide (TMZ) 6. Despite the aggressive multimodal therapy, the recurrence of GBM is frequent, with a median time to recurrence of 9.5 months 7. GBM is characterized by extensive tissue hypoxia, which shapes the phenotypes of glioma stem-like cells (GSCs) 8. Recent single-cell RNA and genome sequencing analyses of GBM have revealed striking intratumoral heterogeneity in hypoxic response and stemness 9. Intratumoral hypoxia contributes to the maintenance of the GSCs by supporting the critical stem cell traits of multipotency, self-renewal, and tumorigenicity 10. In addition to the maintenance of GSCs, hypoxia also contributes to tumor resistance to chemotherapy and radiation 10. A common feature of GBM growth is local recurrence after surgery, and some GBMs recur distally. Indeed, GBM patients with perioperative ischemia are more likely to have distal recurrence 11. A recent study showed that hypoxia could induce the migration and invasiveness of GBM cells 12. Because GSCs contribute to tumor recurrence and resistance to treatments, strategies to eliminate this subpopulation of tumor cells are actively investigated. However, many drugs screening design did not incorporate hypoxia as a critical TME factor.

Protein kinases participate in many signaling pathways, including those involved in cell proliferation, growth, metabolism, apoptosis, and differentiation. Protein kinases are attractive drug targets for malignancy due to their distinct selectivity, pro-oncogenic properties, and a wide selection of readily available kinase inhibitors. Over 50 kinase inhibitors are approved for clinical use by the US Food and Drug Administration, and many others are in clinical development. Molecular crosstalk between HIFs and protein kinases is indispensable for cancer cells' response to hypoxia. While aberrant kinase activities are common in many cancers, hypoxia can further disrupt cellular kinome network to increase the robustness and resilience of oncogenic pathways. HIFs have been shown to activate SAPK, MAPK, and PI3K signaling pathways 13-15. Conversely, various receptor tyrosine kinases, PI3K/Akt, JAK/STAT, and RAS/MEK/ERK pathways augment the stabilization and downstream functional changes of HIFs 16. Hence, targeting hypoxic-dependent kinase signaling hubs can attenuate survival advantages conferred by hypoxic adaptation.

In this study, we employed a “bench-to-bedside and back” approach to identify key signaling conduits exploited by GPCs in hypoxia and reveal opportunities for repurposing kinase inhibitors, currently at different phases of clinical trials. The use of patient-derived GPCs and xenografts in our kinase inhibitor screen and functional assay facilitated the “bench to bedside” analysis, while the “back again” section involved cross-validation with glioblastoma transcriptomes from public repositories. The full range investigative strategy unveiled new insights into the therapeutic prospect of targeting c-MET/PI3K in tumor hypoxia.

## Methods

### Cell culture

All GPC cell lines, NNI-11, NNI-24, and NNI-31, used in this study were derived from patients' tumors and obtained from the National Neuroscience Institute, Singapore. GBM tumor specimens and associated cell lines from the National Neuroscience Institute (NNI) were obtained with informed consent and de-identified in accordance with the SingHealth Centralised Institutional Review Board A. NNI-11 and NNI-24 were cultured in DMEM (Hyclone, UT, USA) with 10% fetal bovine serum (FBS) (Hyclone). The NNI-11, NNI-24, and NNI-31 were cultured in a serum-free growth medium as reported previously 17. The profiling information of the cell lines is described in **Table S1**. All cell lines were maintained in a humidified 37 °C incubator containing 5% CO_2_. Hypoxia was achieved by reducing O_2_ concentration to 1% in a hypoxic chamber.

### Kinase inhibitor screen and viability assay

Kinase inhibitors were obtained from SYNkinase (Australia) and Target Molecule Corp. (MA, USA). The kinase inhibitors, concentrations, and drug targets used in the study are described in **Suppl. File 1**. The cells were treated with kinase inhibitors in normoxic or hypoxic conditions for 72 hours. For cell lines maintained in 10% FBS, they were fixed with 2% formaldehyde and stained with Syto60 fluorescent nucleic acid stain (Invitrogen, CA, USA) to determine the drug cytotoxicity. The area of the stained cell colonies, which represents surviving and proliferative cells, was measured using ColonyArea 18 and expressed as viability. For serum-free GPCs, the viability was determined using alamarBlue Cell Viability Reagent (Thermo Scientific, MA, USA). In both assays, cells treated with kinase inhibitors were normalized to DMSO-treated cells.

### Kinase-kinase pathway analysis and unweighted ranking analysis

Drug targets of the inhibitors which showed enhanced cytotoxicity in hypoxia were visualized and analyzed with Ingenuity Pathway Analysis (IPA) (Qiagen Inc., Germany) and KinMap 19 to map kinase-kinase interaction and identify key kinase signaling pathways involved in the hypoxic adaptation of GBM.

For the ranking analysis, we used an unweighted scoring algorithm similar to Kinase Addiction Ranker 20. Each protein kinase was scored and ranked based on five criteria: direct interaction with HIF-1α (based on IPA network), network topology (number of first-order neighbors from IPA network), and the correlation of their gene expression with GBM patients' overall survival retrieved from Prediction of Clinical Outcomes from Genomic Profiles (PRECOG) 21, The Cancer Genome Atlas Program (TCGA) 22, and Chinese Glioma Genome Atlas (CGGA) 23. The first two criteria were established based on the assumptions that HIF-1α is a master regulator of hypoxic response 24 and that protein kinases with more interacting neighbors are the signaling hubs in the kinase-kinase interactome during hypoxic stress response. The remaining criteria are patients' survival data incorporated into the scoring system to enhance the robustness and clinical relevance. Only GBM patient data from the public depositories were included in the analysis to omit confounding effects from gliomas with lower grades. The data from each criterion were dichotomized into positive and negative groups, except for “network topology” which was quantized into four groups using median, lower, and upper quartiles. For criteria with dichotomous data, positive group was awarded 40 points, and none was given to negative group. For “network topology”, first, second, third and fourth quartiles were given 10, 20, 30, and 40 points, respectively. We sum over the points from each criterion to get a final raw score for each kinase, which was used to rank the kinases. The data of the scoring analysis are available in **Suppl. File 1**.

### Dose-response curve, combination therapy, and drug-drug interaction

Dose-response curves of selected c-MET and PI3K inhibitors, TMZ (SelleckChem, TX, USA), and combined therapy of c-MET inhibitors with TMZ were assessed using Syto60 stain 72-hr post-treatment in normoxia or hypoxia. Drug-drug interaction between c-MET inhibitors and TMZ was examined using the Chou-Talalay method based on the fraction affected (Fa) values and combination index (CI), where CI < 1, CI = 1, and CI > 1 represent synergism, additive effect, antagonism, respectively 25. CI and Fa were computed using Compusyn software (ComboSyn Inc., NJ, USA).

### siRNA knockdown

GPC cells were transfected with siRNA targeting human c-MET from Trifecta RNAi kit (Integrated DNA Technologies Inc., IA, USA) using Lipofectamine 2000 (Invitrogen) for 24 h. Scrambled siRNA was used as a negative control. Transfected cells were incubated at normoxia or hypoxia for 48 h followed by clonogenic assay. To examine the off-target effects of the inhibitors, the viability of c-MET-knockdown GPCs treated with c-MET inhibitors at normoxia and hypoxia for 72 h were examined.

### RNA extraction and qPCR

Total RNA was extracted, and qPCR was performed as previously described 26. The primer sequences for qPCR are in **Table S2**.

### Immunoblotting

Cells were lysed using ice-cold M-PER Mammalian Protein Extraction Reagent (Thermo Scientific) containing Halt Protease Inhibitor Cocktail (Thermo Scientific) and Halt Phosphatase Inhibitor Cocktail (Thermo Scientific). Far-infrared immunoblotting was performed. Protein bands were visualized with the Odyssey^®^CLx Infrared Imaging System (LI-COR Biosciences) and quantified using ImageJ. The effect of tivantinib on α-tubulin polymerization was examined with immunoblotting as described previously 27. All primary antibodies used in this study were from Cell Signaling Technology (MA, USA), except HIF1α (ab179483) from Abcam (UK), GAPDH (0411) from Santa Cruz Biotechnology (TX, USA), and α- (12G10), and β-tubulin (E7) from Developmental Studies Hybridoma Bank (IA, USA).

### Intracellular ROS measurement

Treated cells were stained with CellROX Deep Red Reagent (Life Technologies, CA, USA). The cells were trypsinized, and fluorescence signals were detected at 0, 12, 24, and 48 h post-treatment with BD Accuri C6 Cytometer (BD Biosciences, CA, USA).

### Apoptosis assay and live/dead cell imaging

Apoptotic cells were stained with propidium iodide (Sigma-Aldrich) and Annexin V-FITC (Miltenyi Biotec, Germany) and detected using BD Accuri C6 Cytometer. Live and dead cells were distinguished by staining with propidium iodide and Hoechst 33342 (Invitrogen) and imaged using Ju LI Stage fluorescent microscope (NanoEnTek Inc., South Korea).

### Animal experiment

2×10^6^ NNI-24 cells were resuspended in Matrigel (Corning, NY, USA) and culture medium and implanted into the dorsal flank of 8-week old male NSG mice. Mice were maintained in a facility with a 12 h dark/light cycle and *ad libitum* access to chow diet and water. The tumor volume was measured weekly using a digital caliper and calculated using the following formula: (width^2^ x length)/2. When tumor volume reached approximately 100 mm^3^, the mice were randomized to six groups, which were given one-time intratumoral injection of saline (vehicle control), TMZ (25 mg/kg), PF042178903 (1 mg/kg), TMZ (25 mg/kg) + PF04217903 (1 mg/kg), Tivantinib (50 mg/kg), or TMZ (1 mg/kg) + Tivantinib (50 mg/kg), respectively. For histopathology and intratumoral cytokine profiling assays, the tumor xenografts were harvested at day three post-injection. Tumor weight was determined at week three post-injection. All procedures were performed according to the Nanyang Technological University's Institutional Animal Care and Use Committee guidelines (A0321, A0324, A19032, A19034).

### Histological and immunofluorescence staining

Tissues were processed, sectioned, and stained as previously described with minor modifications 26. Heat antigen retrieval was performed in sodium citrate buffer (10 mM, 0.05% Tween-20, pH 6) using Aptum Biologics 2100 Antigen Retriever (Aptum Biologics, UK). The sections were blocked with 5% (v/v) fetal bovine serum for an hour and labeled with anti-HIF1α (ab179483; Abcam, UK) and anti-cleaved caspase-3 (Merck Millipore, MA, USA) antibodies at °C overnight. The sections were then treated with Alexa Fluor 680-conjugated secondary antibody and counterstained with DAPI. Microscopic images of the sections were captured with Zeiss Axio Scan.Z1 (Carl Zeiss AG, Germany).

### Inflammatory cytokine profiling

Cytokine profiling of the cell lysates, culture media, and tumor lysates was performed with LUNARIS^TM^ Human 11-Plex Cytokine kit (AYOXXA Biosystems GmbH, Germany) according to the manufacturer's instruction.

### Bioinformatics analysis with public GBM databases

RNAseq data of clinical GBM tumors (n = 37 tumors) from different anatomic structures (i.e., leading edge, infiltrating tumor, cellular tumor, microvascular proliferation, and pseudo-palisading cells around necrosis) were obtained from IVY Glioblastoma Atlas Project 28. Differential gene expression analysis between peri-necrotic and cellular tumor regions was performed with DESeq2 29. Differentially expressed genes (DEGs) are those with log2 Fold Change > ± 1 and false discovery rate < 0.05. Functional enrichment analysis was performed using Ingenuity Pathway Analysis and Gene Set Enrichment Analysis (GSEA) 30. Transcription factor enrichment analysis of the significantly upregulated genes in the peri-necrotic zone was performed with TFEA.ChIP 31. The output was mapped to protein kinases using PhosphoAtlas 32. Single-cell RNA sequencing data were obtained from Darmanis et al. (2017) 33 and analyzed with Seurat 34.

### Statistical analysis

Dependent variables with repeated measures (*e.g.,* tumor volume over time) were analyzed using a mixed-model ANOVA. Dependent variables with two main factors (*e.g.,* dose-response between normoxia and hypoxia) were analysed with a two-way ANOVA. Pairwise comparisons were performed with Sidak correction. Other variables were analyzed with one-way ANOVA, followed by post hoc Tukey's test. P-values < 0.05 indicate statistical significance.

## Results

### Hypoxia modifies tumor kinomic landscapes and reveals c-MET-PI3K signaling pathway needed for hypoxic response in GPCs

A total of 188 small-molecule compounds were screened against 130 different kinases using a cell-based viability assay. This functional assay directly measures GPCs, NNI-11, and NNI-24, cultured under normoxia and hypoxia (1% O_2_). The compounds screened included 39 U.S. FDA-approved drugs, 45 compounds which are in active clinical development (5 in Phase I, 26 in Phase II, and 14 in Phase III), 42 compounds that were dropped from the clinical pipeline, 61 compounds in preclinical testing, and 1 compound withdrawn from the market (**Suppl. File 1**). The resultant cell viability data were subjected to unbiased hierarchy clustering analysis and presented in a heatmap (**Figure S1A; Suppl. File 1**).

An inhibitor is considered cytotoxic when it reduced GPCs' viability by 10% relative to DMSO treatment. Most inhibitors (62.8%; 118 inhibitors), including the vehicle, DMSO, were non-cytotoxic at the tested concentration in both normoxic and hypoxic environments (**Figure S1B-C**). Thirty-six inhibitors (19.1%) were cytotoxic in normoxia, while 29 inhibitors (15.4%) were cytotoxic in hypoxia. Astoundingly, only 5 out of the 188 kinases inhibitors (2.7%) were cytotoxic to GPCs in normoxia and hypoxia, of which KX2-391 (normoxia: 63% viability; hypoxia: 49.5%) and Staurosporine (normoxia: 51%; hypoxia: 32%)] were more hypoxic-selective whilst AT-7519 HCl (normoxia: 31%; hypoxia: 66%) and BMS387032 HCl (normoxia: 56%; hypoxia: 80.5%)] were more normoxic-selective. Lenvatinib (normoxia: 88%; hypoxia: 89.5%) showed comparable cytotoxicity in both conditions. Clearly, the profound changes of GPCs' drug response and the low number of effective kinase inhibitors indicate the extensive modifications of cellular kinome activities under hypoxia.

**Figure 1:**
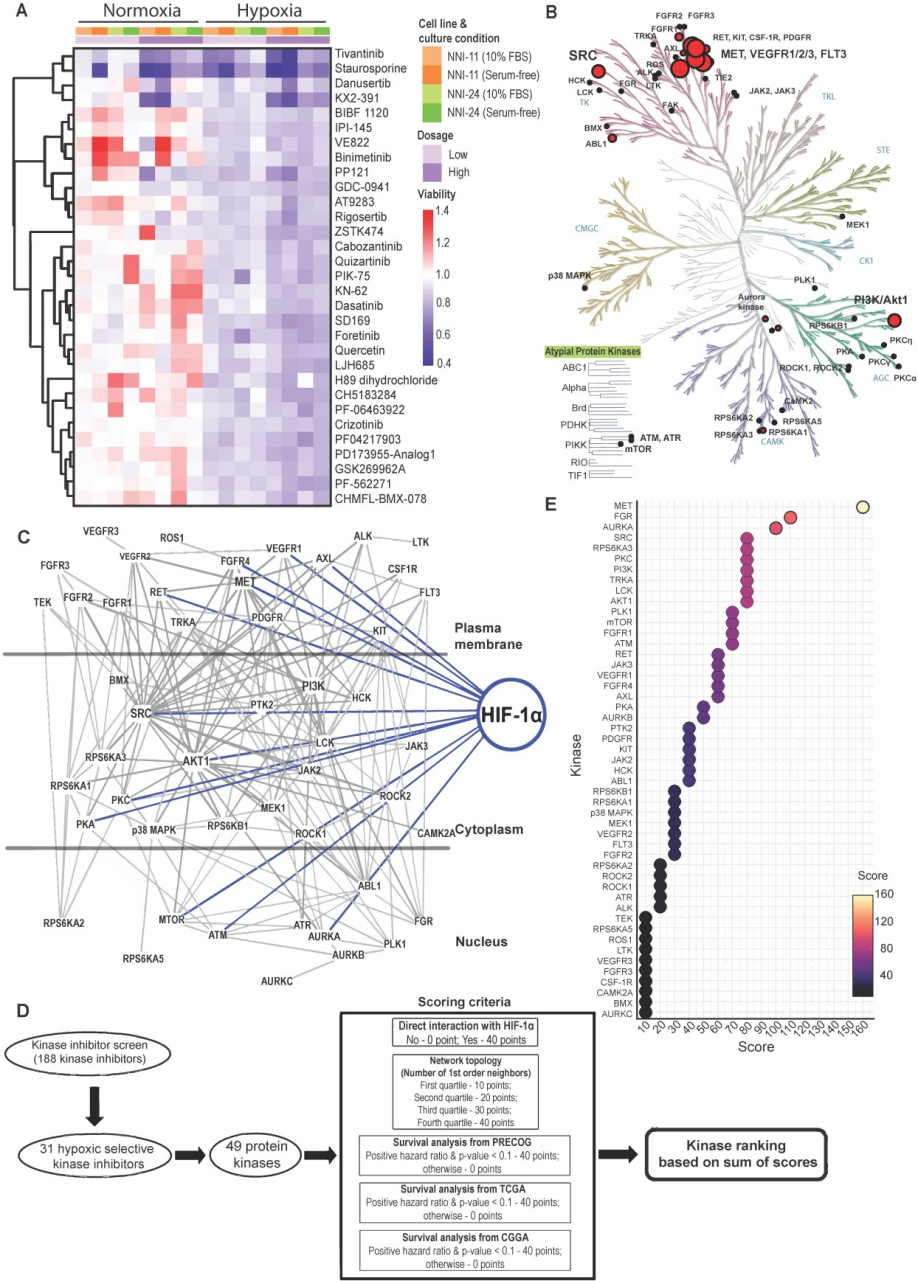
** Kinome landscape in hypoxic adaptation of GPCs. (A)** Selected kinase inhibitors with hypoxia-selective cytotoxicity in GPCs. The cell viability (relative to DMSO-treated cells) is color-coded. Blue color indicates reduced viability, while red color indicates increased viability compared to DMSO-treated cells. **(B)** Protein kinases targeted by hypoxic-selective small-molecule inhibitors. Implicated kinases are highlighted in red circles. Sizes of the circles indicate the number of shortlisted inhibitors targeting the kinases (i.e., a larger circle implies a higher number of inhibitors targeting the kinase; min: 1 inhibitor, max: 5 inhibitors). **(C)** Gene connectivity network generated based on the molecular targets of hypoxic selective inhibitors using Ingenuity Pathway Analysis. Dark grey lines indicate the kinase-kinase interactions, while blue lines indicate HIF-1α-kinase interactions. **(D)** Workflow and scoring criteria used in the unweighted ranking analysis. Protein kinases are ranked based on the connectivity network and the correlation between their expressions and GBM patients' overall survival. **(E)** Kinase ranking based on the unweighted sum of scores of each protein kinase. Higher ranks predict a more important role of the kinase in tumor hypoxia of GPCs based on the network analysis and clinical data.

The kinase inhibitor screen revealed 31 hypoxic-selective inhibitors whose molecular targets could be crucial for hypoxic response and survival in GPCs. To study if the hypoxic-selectivity of these inhibitors is affected by stemness and culture conditions, we performed another screen using GPCs maintained in either 10% FBS or serum-free conditions (**Figure 1A; Suppl. File 1**). The cytotoxicity effects of the hypoxic-selective inhibitors exhibited a dose-dependent trend and were comparable between GPCs grown at different conditions. The results suggest that the hypoxic selectivity is unaffected by the stemness of the tumor cells. Furthermore, they also highlight a dominant role for hypoxia in the TME on GPCs' response to drugs. The drug targets of hypoxic-selective inhibitors were then mapped out and amounted to 49 protein kinases (**Figure 1B**). Certain kinases, notably PI3K/Akt, Src, c-MET, VEGFR1/2/3, and FLT3 were over-represented by multiple inhibitors, implying that these targets are likely to be true positives and key mediators of tumor hypoxic adaptation. We also analyzed the protein kinases with the IPA data repository to unravel the kinome network, differentially altered by hypoxia in GPCs (**Figure 1C**). The interaction map revealed that Src and PI3K/Akt pathways are highly intertwined with other kinases, forming signaling hubs in the GPC's response to hypoxic stress. Several upstream receptor tyrosine kinases, namely c-MET, VEGFR1, AXL, FGFR4, and RET, interacted directly with the signaling hubs and HIF-1α, further supporting the pivotal roles of Src and PI3K/Akt signaling in hypoxia response. To rank and shortlist kinase candidates using information from the connectivity network, we employed a ranking algorithm similar to Kinase Addiction Ranker 20. All 49 protein kinases targeted by the 31 hypoxic-selective inhibitors were scored individually according to their interaction with HIF-1α and network topology in addition to the predicted GBM patients' survival based on their expression status retrieved from PRECOG 21, TCGA 22, and CGGA 23 to strengthen the robustness and clinical relevance of the analysis (**Figure 1D**). The sum of scores from each category was used to rank the protein kinases (**Figure 1E**). c-MET emerged as the top-ranked kinase. Src and PI3K/Akt, identified as signaling hubs in tumor hypoxic response, were also among the highest-ranking kinases. Importantly, c-MET, Src, PI3K/Akt share mutual interaction and with HIF-1α, clearly highlighting that the triad could be a targetable signaling conduit to undermine hypoxic adaptation by GPCs.

### c-MET-dependent hypoxic response is mediated by antioxidant effect of PI3K/Akt pathway

**Figure 2:**
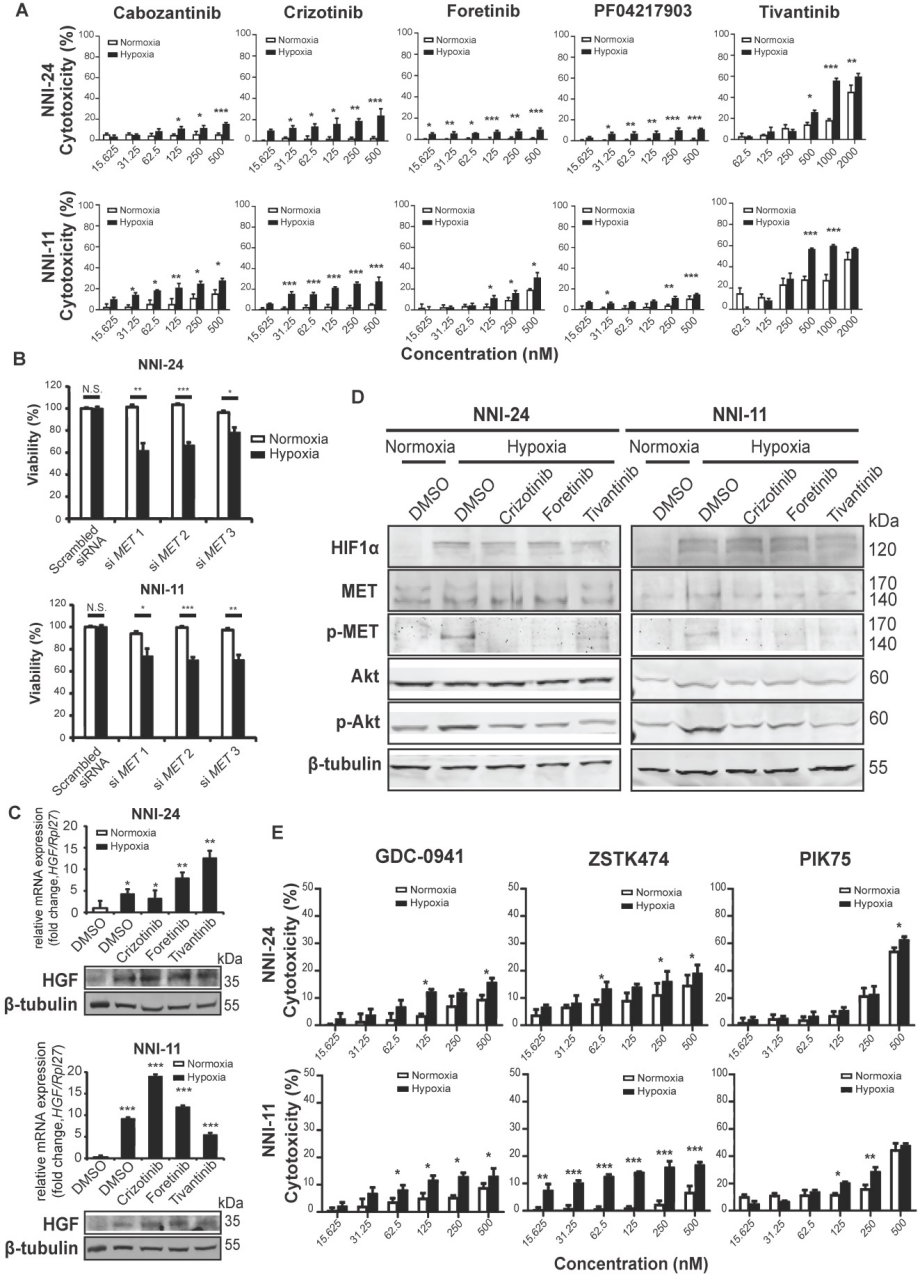
** c-MET-dependent hypoxic response is mediated by PI3K/Akt pathway. (A-B)** Cell viability assays of NNI-24 and NNI-11 treated with increasing concentrations of c-MET inhibitors (A) or whose c-MET expression was suppressed by three different siRNAs (B) and exposed to normoxic and hypoxic conditions. Data are represented as mean±SD from n = 3 replicates. * p < 0.05, ** p < 0.01, *** p < 0.001. **(C)** Relative mRNA (top panel) and protein (bottom panel) expression of *HGF* in NNI-24 and NNI-11 treated with either crizotinib (500 nM), foretinib (500 nM), or tivantinib (1000 nM) and exposed to hypoxia. For mRNA expression, *Rpl27* was used as the endogenous reference gene. Data are represented as mean±SD from n = 3 replicates. * p < 0.05, ** p < 0.01, *** p < 0.001 compared to normoxia group. **(D)** Representative immunoblots of indicated proteins in NNI-24 and NNI-11 treated with crizotinib (500 nM), foretinib (500 nM) and tivantinib (1000 nM) and exposed to hypoxia. β-tubulin which serves as a loading control was from the same samples. **(E)** Cell viability assays of NNI-24 and NNI-11 treated with increasing concentrations of PI3K inhibitors and exposed to normoxic and hypoxic conditions. Data are represented as mean±SD from n = 3 replicates. * p < 0.05, ** p < 0.01, *** p < 0.001 compared to corresponding drug concentration in normoxia.

Although most kinase inhibitors have been developed against a specific kinase target, many inhibitors often inhibit multiple kinases in key signaling pathways and exhibit off-target effects. To mitigate such limitations, we examined the dose-response of five structurally different c-MET inhibitors, namely cabozantinib, crizotinib, foretinib, PF04217903, and tivantinib, as well as knockdown of c-MET on the viability of GPCs in normoxic and hypoxic conditions (**Figure 2A**-**B**, **S1D**). The cytotoxicity effects of c-MET inhibitors were markedly increased under hypoxia. The hypoxic selectivity of crizotinib, foretinib, and tivantinib was consistent in NNI-24 and NNI-11. In line with the findings from c-MET inhibitors, the knockdown of c-MET also conferred hypoxic-selective cytotoxicity. Altogether, these observations indicate that the attenuation of c-MET compromises the survival of GPCs in a hypoxic environment.

To understand how hypoxia affects c-MET signaling in GPCs, we first examined the expression of hepatocyte growth factor (HGF), the natural ligand of c-MET. We observed a significant increase in the expression of HGF in GPCs under hypoxia than in normoxia (**Figure 2C**). Likewise, hypoxia also enhanced phosphorylation of c-MET following HIF-1α upregulation (**Figure 2D**). While c-MET inhibitors had no impact on HGF expression, the phosphorylation of c-MET was effectively diminished by crizotinib, foretinib, and tivantinib, leading to the inactivation of c-MET signaling. To assess the off-target effects of these inhibitors, we treated c-MET-knockdown GPCs with the inhibitors (**Figure S1E**). Crizotinib and foretinib did not induce further cytotoxicity, indicating that their hypoxic selectivity is attributable to c-MET inhibition. Surprisingly, tivantinib induced an additional 10-20% viability reduction to c-MET-knockdown GPCs in a non-hypoxic-selective manner, suggesting that the off-target of tivantinib has significant cytotoxicity. Tivantinib has been known to inhibit tubulin polymerization 27, so we performed immunoblots to check polymerized microtubules in tivantinib-treated GPCs. Our results also revealed that tivantinib reduced α-tubulin level in the insoluble fraction of cell lysates independent of the oxygen environment (**Figure S1F**), which suggests a disruption of α-tubulin assembly. The finding may explain the discrepancy in drug efficacy between tivantinib and other c-MET inhibitors.

The kinase-kinase interaction map suggests that Src and PI3K/Akt are the key downstream signaling hubs of c-MET activation (**Figure 1C**). However, Src phospho-activation was unaffected by hypoxia and c-MET inhibitors, even though Src inhibitors like KX2-391, bosutinib, and dasatinib did exert hypoxic-dependent cytotoxicity dose-dependently, especially in NNI-24 (**Figure S2A-B**). Hence, the activation of Src may not be strictly c-MET-dependent in tumor hypoxia of GPCs. In contrast, the protein expression and phosphorylation of Akt were elevated under hypoxia and diminished by c-MET inhibitors (**Figure 2D**). Exposure to hypoxia also increased the number of dead GPC cells treated with PI3K inhibitors like GDC-0941, ZSTK474, and PIK75 in a dose-dependent manner (**Figure 2E**). Additionally, the hypoxic selectivity of c-MET and PI3K inhibitors was again validated in another patient-derived cell line, NNI-31 tumoroids, cultured in a serum-free environment. As anticipated, tivantinib (c-MET) and PIK75 (PI3K) inhibitors triggered apoptosis in hypoxic GPCs even at a low concentration (**Figure S2C-D**). We also examined the activity of Ras-Raf-MEK-ERK pathway, another key signal transducer of c-MET, and found that it was suppressed in hypoxia. Expectedly, the treatment of c-MET inhibitors neither affected the expression nor phosphorylation status of B-Raf and MEK1/2 (**Figure S2A**). The results indicate that MEK-ERK activation may serve as an early responder to hypoxia but has limited functionality in prolonged hypoxia 35, 36. In summary, our observations suggest that PI3K is the major signaling hub downstream of c-MET in the hypoxic response by GPCs.

**Figure 3:**
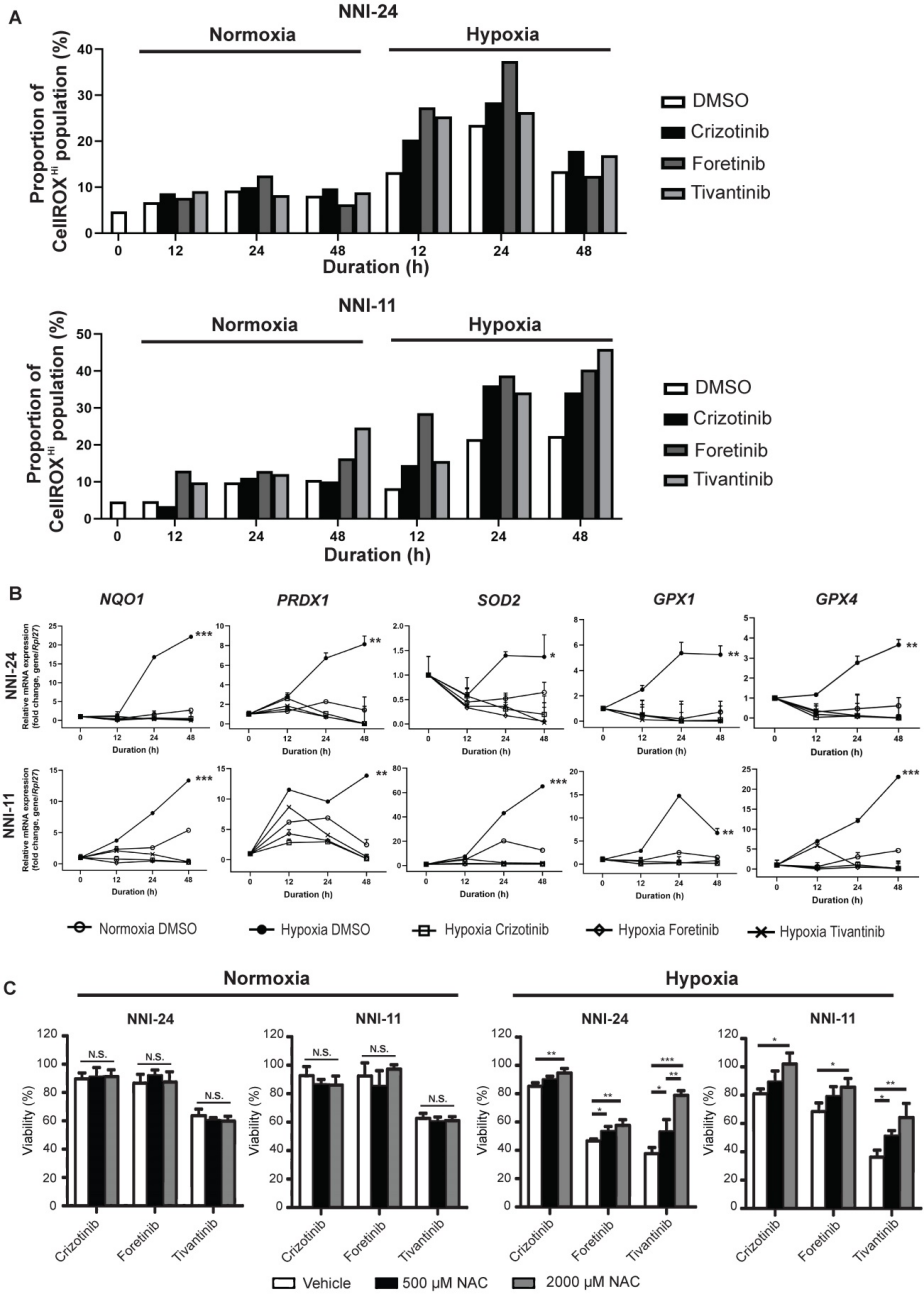
** c-MET inhibition disrupts antioxidant mechanism in hypoxia. (A)** Percentage of NNI-24 (top) and NNI-11 (bottom) cell population with high intracellular ROS (CellROX^Hi^) when treated with crizotinib (500 nM), foretinib (500 nM), and tivantinib (1000 nM) for 0, 12, 24, and 48 h in normoxia and hypoxia. **(B)** Relative mRNA expression of antioxidant genes in NNI-24 and NNI-11 treated as described in (A) for 0, 12, 24, and 48 h in hypoxia. *Rpl27* was used as the endogenous reference gene. Data are represented as mean±SD from n = 3 replicates. * p < 0.05, ** p < 0.01, *** p < 0.001 compared to other groups. **(C)** Cell viability of NNI-24 and NNI-11 treated as described in (A), in the presence or absence of N-acetyl cysteine (NAC) in normoxia and hypoxia. NAC was used at 500 and 2000 µM. Data are represented as mean±SD from n = 3 replicates. * p < 0.05, ** p < 0.01, *** p < 0.001, N.S. denotes non-significant.

Hypoxia alters intracellular reactive oxygen species (ROS) balance, a prosurvival signal in GPC self-renewal, and proliferation 37. PI3K/Akt signaling is activated by hypoxia to mitigate oxidative stress caused by metabolic reprogramming 38. Thus, we questioned if the inhibition of c-MET-PI3K cascade in GPCs under hypoxia, resulting in increased apoptosis, can be attributed to oxidative catastrophe. Indeed, the hypoxic environment escalated the population of GPCs with high intracellular ROS levels (**Figure 3A, S3, S4**). Cellular response to oxidative stress invokes the upregulation of Nrf2-regulated antioxidant genes like* NQO1*, *PRDX1*, *SOD2*, *GPX1*, and *GPX4* (**Figure 3B**). Treatment with c-MET inhibitors increased the high-ROS GPC population in hypoxia. In both GPCs, the population with high ROS levels peaked at 24 h. In NNI-11, the population plateaued from 24 to 48 h, but in NNI-24, such population declined at 48 h, likely because of oxidative stress-induced cell death (**Figure 3A, S3, S4**). Clearly, the inhibition of c-MET by small molecule inhibitors further exacerbated ROS accumulation, outstripping the antioxidant capacity of the cells. Such a buildup of intracellular ROS likely due to impaired Nrf2-mediated gene regulation, as demonstrated by the persisted suppression of Nrf2-regulated antioxidant genes upon c-MET inhibitor treatment (**Figure 3B**). Moreover, the supplementation of a strong antioxidant, N-acetyl cysteine, dose-dependently rescued GPCs from the cytotoxic effect of c-MET inhibitors in hypoxia, but not normoxia (**Figure 3C**). The results further reinforce the involvement of the antioxidant mechanism in hypoxic adaptation. In summary, c-MET/PI3K/Akt signaling pathway is vital for GPCs to alleviate oxidative stress in hypoxic TME.

### c-MET inhibitors synergize with temozolomide under hypoxia

**Figure 4:**
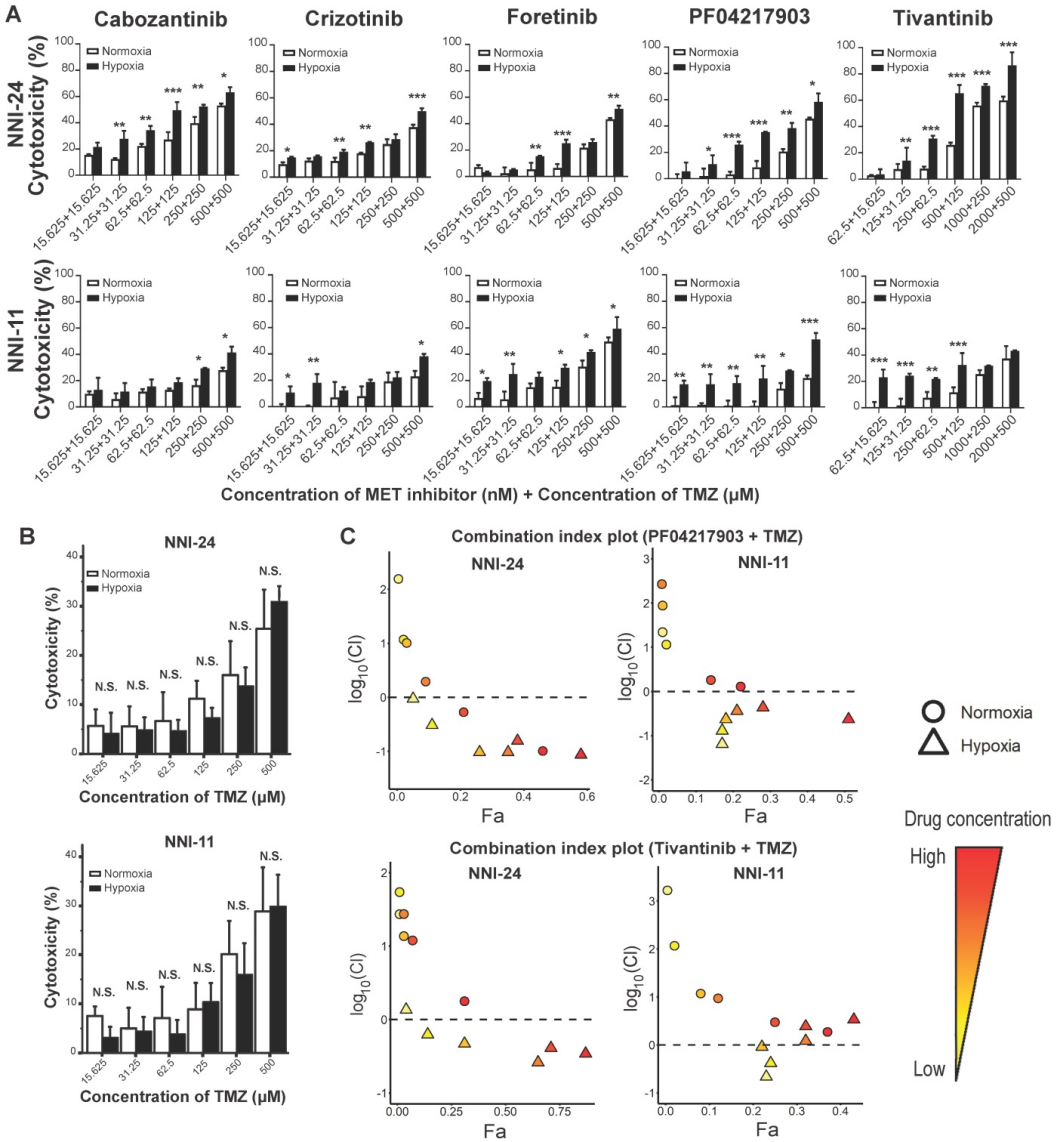
** Hypoxia-dependent drug synergism between c-MET inhibitors and TMZ show enhanced anti-tumor effect in mouse GPC xenografts. (A-B)** Cell viability assays of NNI-24 and NNI-11 treated with combined therapy of c-MET inhibitors and TMZ (A) or TMZ alone (B) at increasing concentrations and exposed to normoxic and hypoxic conditions. Data are represented as mean±SD from n = 3 replicates. * p < 0.05, ** p < 0.01, *** p < 0.001 and N.S. denotes non-significant when compared to corresponding drug concentration in normoxia. **(C)** Fa-log_10_(Cl) plots of NNI-24 and NNI-11 treated with combined therapy of PF04217903 or tivantinib with TMZ at increasing concentrations and exposed to normoxic and hypoxic conditions. Drug combinations with log_10_(Cl) < 0 (dotted line) indicate synergism. Increasing color intensity corresponds to increasing concentrations of the drugs.

TMZ is the current standard-of-care chemotherapeutic drug for newly-diagnosed GBM patients 6. Therefore, it is of interest to assess the efficacy of combined therapy between c-MET inhibitors and TMZ. Hypoxic selectivity of c-MET inhibitors was preserved even in combination with TMZ in a dose-dependent trend (**Figure 4A**), while TMZ alone did not exhibit any hypoxic selectivity (**Figure 4B**). Based on the fraction affected (Fa)-combination index (CI) plots computed using Compusyn software, most c-MET inhibitors synergized favorably with TMZ under hypoxia, but not in normoxia (**Figure 4C**, **S5A**). Among the five c-MET inhibitors, PF04217903 and tivantinib exhibited the most notable drug synergism with TMZ under hypoxic conditions (**Figure 4C**).

**Figure 5:**
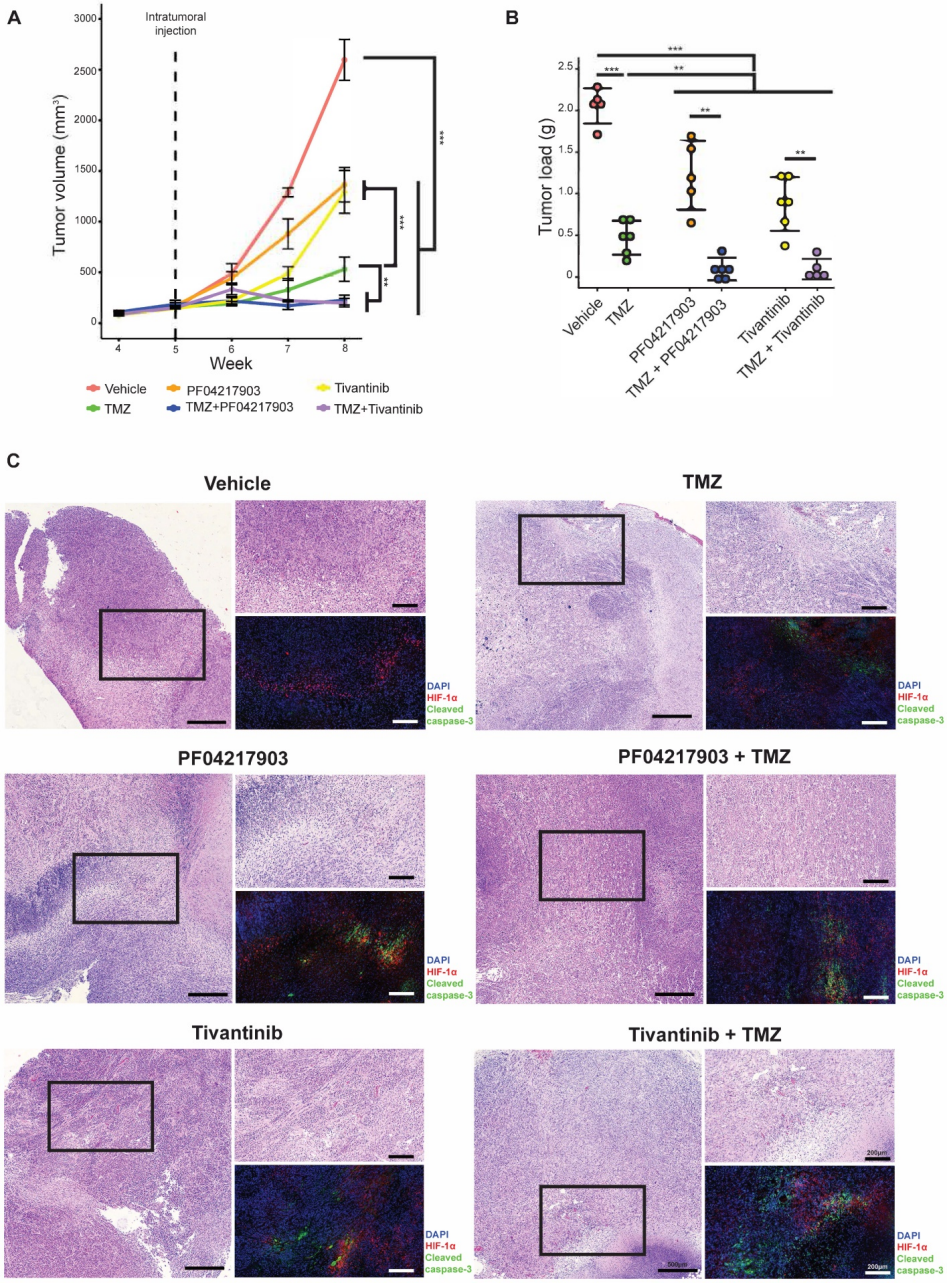
** Tumor analysis of GPC xenograft models. (A)** Growth of GPC xenografts in mice treated with vehicle, TMZ, PF04217903, PF04217903+TMZ via intratumoral injection. Data are represented as mean±SD from n = 5-6 biological replicates. * p < 0.05, ** p < 0.01, *** p < 0.001 between groups. **(B)** Tumor load of GPC xenografts in mice treated with vehicle, TMZ, tivantinib, tivantinib+TMZ via intratumoral injection. Data are represented as mean±SD from n = 5-6 biological replicates. * p < 0.05, ** p < 0.01, *** p < 0.001 between groups. **(C)** Representative images of GPC xenografts at Day 3 post-injection. Immunofluorescence staining for HIF-1α (red) and cleaved caspase-3 (green) while the nuclei were counterstained with DAPI (blue). Hematoxylin and eosin (H&E) staining was performed with other sections from the same tumor xenografts. Scale bars are 500 µm and 200 µm for wide and magnified fields, respectively.

Next, we investigated the drug synergism *in vivo*. As expected, co-treatment of TMZ with either PF04217903 or tivantinib effectively attenuated the growth of NNI-24 tumor xenograft and reduced tumor load (**Figure 5A**-**B**). We also performed immunofluorescence staining of HIF-1α and cleaved caspase-3 to distinguish the extent of apoptotic death in the hypoxic and non-hypoxic regions of tumor xenografts (**Figure 5C**). The immunofluorescence images revealed that hypoxic areas were pervasive in GPC xenografts. Vehicle-treated xenografts had minimal apoptosis. TMZ induced cellular apoptosis largely restricted to HIF-a negative regions, i.e., non-hypoxic.

Interestingly, the c-MET inhibitors PF04217903 and tivantinib induced activation of caspase-3 in the hypoxic regions. As a result, apoptosis was notable in both hypoxic and non-hypoxic areas of the tumors with co-administration of TMZ with c-MET inhibitor. Thus, our *in vivo* results showed that inhibition of c-MET fortified TMZ cytotoxicity in the hypoxic environment, significantly mitigating tumor xenograft growth.

### Clinical validation of hypoxic kinase inhibitor screen with GBM transcriptomes

**Figure 6:**
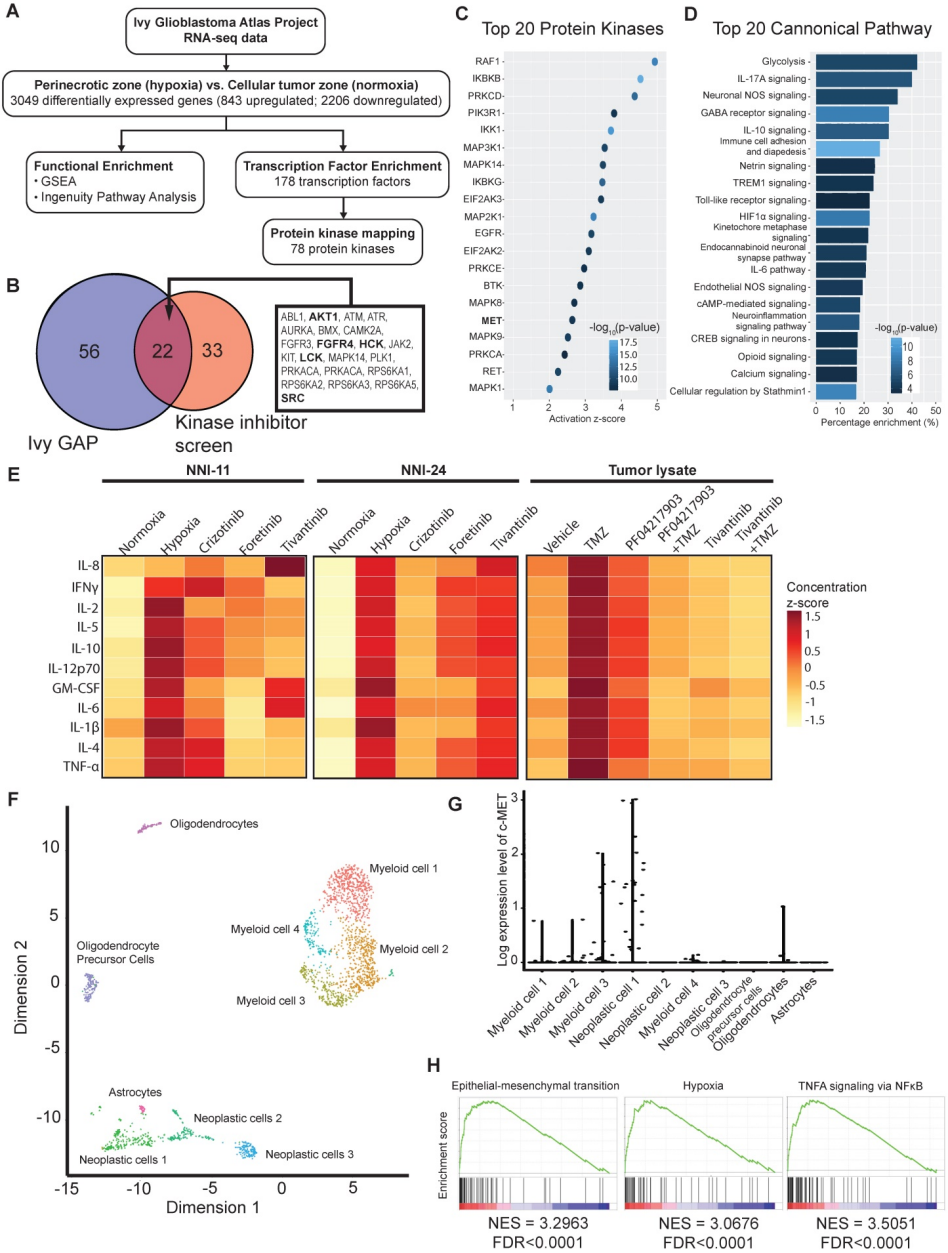
** Bulk and single-cell transcriptomes of clinical GBM tumors reveal susceptibility of aggressive tumor subpopulation to c-MET inhibition. (A)** Analytical analysis pipeline of RNA-seq data (Ivy GBM Atlas Project) to identify enriched pathways, transcription factors, and protein kinases in the peri-necrotic and hypoxic regions of GBM specimens. **(B)** Venn diagram illustrates the hypoxic-selective protein kinases derived from Ivy GBM Atlas Project and our kinase inhibitor screen. Twenty-two common kinase targets are listed, and those shown in bold are downstream targets of or kinases that have direct protein-protein interaction with c-MET. **(C)** Top 20 activated protein kinases predicted by Ingenuity Knowledge Base repository based on DEGs of the peri-necrotic zone compared to cellular tumor zone in clinical GBM specimens. **(D)** Top 20 enriched pathways predicted by Ingenuity Knowledge Base repository based on DEGs of the peri-necrotic zone compared to cellular tumor zone in clinical GBM specimens. **(E)** Multiplex cytokine profiling of the cell lysates from NNI-11 (left panel), NNI-24 (middle panel), and tumor lysates from GPC xenografts at Day 3 post-injection (right panel). For *in vitro* cell assays (n = 3 replicates), the cells were treated with crizotinib, foretinib, and tivantinib and exposed to hypoxia. Vehicle (DMSO)-treated cells exposed to normoxia and hypoxia serve as the control groups. For *in vivo* experiments, mice bearing GPC xenografts (n = 5-6 biological replicates) were given the indicated treatments. The cytokine concentrations are color-coded and expressed in the concentration z-score. **(F)** t-SNE plot of the re-analyzed single-cell transcriptomes of clinical GBM tumors obtained from GSE84465. Different cellular subpopulations are color-coded and annotated. **(G)** Log-expression level of c-MET in different cellular subpopulations based on the single-cell transcriptomes. **(H)** Highly activated pathways in the neoplastic subpopulations with c-MET overexpression compared to those with low c-MET expression.

Our above findings suggest intratumoral heterogeneity with respect to hypoxia-selective drug susceptibility. Due to the paucity of fresh GBM biopsies, we leveraged on the RNA-seq data of clinical GBM tumors from Ivy GBM Atlas Project 28 to validate our kinase inhibitor screen and *in vivo* results. We found 843 upregulated and 2206 downregulated genes (3049 DEGs), of which the former were mapped to 178 transcription factors, and subsequently, 78 protein kinases were mapped to the hypoxic regions of GBM (**Figure 6A**). Twenty-two out of the 78 enriched kinases intersect with the hypoxic-selective kinases from our screen, especially the highest-ranking targets, i.e., AURKA, SRC, RPS6KA3, etc. (**Figure 1E, 6B**). The activation of c-MET and its downstream kinase targets are anticipated (**Figure 6B-C**) based on Ivy GBM Atlas Project and Ingenuity Knowledge Base repositories. Interestingly, proinflammatory mediators and pathways are highly enriched in the hypoxic zone (**Figure 6D, Suppl. File 2**). Cytokine profiling also confirmed the increased expression and secretion of proinflammatory cytokines in cells exposed to hypoxia and tumor xenografts treated with and without TMZ treatment, whereas the treatment with c-MET inhibitors significantly resolved the proinflammatory response (**Figure 6E, S5B**). Single-cell transcriptomes of GBM tumors from Darmanis et al. (2017) revealed that c-MET overexpression was only present in a subpopulation of the neoplastic GBM cells that were more hypoxic, inflamed, stem-like, and metastatic (**Figure 6F-H, Suppl. File 3**). This highly invasive subpopulation could be more vulnerable to the adjuvant therapy with c-MET inhibitors in clinical settings.

## Discussion

Hypoxic TME is a hallmark of many cancers. As normal tissues are generally unaffected by hypoxia, exploiting this distinct feature of TME may lead to highly specific anti-tumor treatments. Tumor hypoxia is a pivotal modifier of tumor chemosensitivity, contributing to treatment failure 39 and even a high drug attrition rate in the clinical pipeline 40. Still, very few studies proactively consider this variable in their preclinical drug screens despite knowing the pharmacological interference of hypoxia. In this context, GBM is notoriously refractory to therapy and highly infiltrative owing to its stem cell-like properties enacted mainly by the high intra-tumoral hypoxia 8. Such reliance on hypoxia-induced survival advantages by GBMs makes them vulnerable to anti-neoplastic agents that switch off their adaptive hypoxic responses.

Hypoxia modulates the activity of a series of kinase inhibitors on GPCs, with only a handful of small molecule inhibitors retaining their cytotoxicity irrespective of the oxygen environment 41. Indeed, hypoxic stress is a strong selection pressure that can lead to the emergence of subpopulations of viable cells with drastically altered tumor kinome landscapes, although the implicated protein kinases in different tumor cells may vary 42, 43. As a result, small molecule inhibitors that are cytotoxic in normoxia may lose their potency in hypoxia simply because of the change in predominant protein kinases. Furthermore, tumor hypoxia profoundly influences the local physiological conditions, such as the onset of acidosis and redox imbalance 44, 45. The concentration of oxidative and reductive species and pH can interfere with the physicochemical properties of small molecule inhibitors, modifying important functional groups, drug conformation, and even drug stability, which collectively leads to differential pharmacodynamic profiles between normoxia and hypoxia 46, 47. Essentially, our results from the kinase inhibitor screen highlight a substantial modifying effect of oxygen environment on the effectiveness of small molecule inhibitors, thus warranting the need to consider hypoxia in cancer drug development.

Combining the kinase inhibitor screen, network analysis, and an unweighted scoring algorithm that includes clinical data, we identify c-MET-PI3K pathway as an essential and targetable signaling hub that is vital for GPCs' survival in hypoxic TME. Our interrogation of bulk and single-cell transcriptomes from clinical GBM specimens also supports the involvement of c-MET signaling in invasive tumor behaviors. The blockade of c-MET signaling, on the other hand, successfully weakened GPCs, resulting in increased apoptosis. Co-administration of c-MET inhibitors and TMZ demonstrated favorable drug synergism that significantly attenuated GPC tumor xenografts' growth. These findings confirm that the c-MET signaling network can serve as a potential oncogenic target to exploit hypoxic TME in GBM.

In another study, abolishing HGF/c-MET axis with antisense transgenes also substantiated GBM response to radiotherapy in a synergistic manner 48. Such therapeutic synergy offered by c-MET suppression not only explains why superior sensitivity to radio- and chemotherapy is seen in GBM patients with low-c-MET expression 49, 50 but also provides empirical support for the clinical development of c-MET inhibitors and monoclonal antibody (onartuzumab) as adjuvant therapies for GBM 51. A recent phase Ib clinical study reported a favorable safety profile and efficacy of multimodal GBM therapy with crizotinib, TMZ, and radiotherapy, supporting the feasibility of incorporating c-MET inhibitors into the current GBM treatment regime 52. In summary, c-MET is a key adaptive player under hypoxia. Targeting c-MET sensitize GBM to the cytotoxic effect of TMZ and potentiates the overall treatment outcome, making it a promising adjuvant candidate for GBM therapy.

Targeting c-MET activity with small molecule inhibitors adversely disrupts tumor proliferation, neovascularization, and distal metastasis across different cancer types, including gastric, breast, hepatic, pancreatic, and lung carcinomas, suggesting that HGF/c-MET axis is a crucial survival expedient for tumor cells in hypoxic TME 53. We revealed a non-canonical c-MET-PI3K-mediated antioxidant mechanism in GPCs upon exposure to hypoxia. c-MET inhibitor led to an accumulation of intracellular ROS in GPCs that outstripped the endogenous antioxidant mechanism, resulting in apoptotic cell death in a hypoxic-dependent manner. The beneficial effect may be attributable to the anti-inflammatory effect of c-MET attenuation considering the crucial roles of c-MET induced TME inflammation and cytokine signaling in many pro-tumorigenic processes and cancer cell survival 54, 55. Our finding is in line with another recent study that demonstrated that c-MET activation triggered the nuclear localization of Nrf2 and its downstream effector, heme oxygenase-1, to resolve drug-induced oxidative stress in renal tumors 56. Coincidentally, PI3K/Akt pathway also acts as an activator of Nrf2 transcription and nuclear translocation to modulate redox homeostasis and enhance cancer survival during carcinogenesis 57, 58. Hence, cancer cells can actuate c-MET-PI3K-Nrf2 signaling cascade upon hypoxia exposure to mitigate the imminent threat from oxidative stress buildup while more complex defense machineries (i.e., angiogenesis and epithelial-to-mesenchymal transition) take effect to preserve their long-term survival. Thus, the inhibition of c-MET effectively arrests the short-term adaptive response (i.e., antioxidant functionality) and undermines long term responses against redox disturbance from hypoxia.

Our findings are established on a drug screen that used IC50 values derived from cell-free assays and optimized to detect each inhibitor's differential effectiveness between normoxia and hypoxia. As the reported IC50 values for every inhibitor span across a considerable range (nM to µM), the resultant cytotoxicity read-outs should not be used to compare the efficacy between different inhibitors directly. Furthermore, the relationship between small-molecule inhibitors and their drug targets is a complex many-to-many association, rather than a simple one-to-one association. An inhibitor can have multiple targets, and different inhibitors share one or multiple common targets. The drug concentration adds another layer of complexity as target specificity is dose-dependent. A high throughput inhibitor screen poses practical challenges to eliminate the off-targets of every inhibitor. Hence, in our screening informatics strategy, all known targets of the hypoxic selective inhibitors were included in the subsequent analyses. The ranking algorithm also helps to sieve the information and extract the true targets based on network analysis and clinical data prediction. True targets that were underrepresented by our drug panel will be missed out in our approach. However, high-ranking kinases from our approach are experimentally shown to be hypoxic selective with supports from biological mechanisms and clinical prediction, and hence are highly likely to be true targets that are worth further investigation.

Another limitation is associated with the use of flank implantation of patient-derived tumor cells in mice, which is unable to fully recapitulate the central nervous system environment. However, to our knowledge, none of the five c-MET inhibitors used in our study has been reported to cross blood-brain barrier (BBB) effectively, thus limiting our selection to subcutaneous xenograft models. Drug delivery across the BBB remains one of the most challenging fields for pharmaceutical and biotechnological products. Our findings can spur the development of c-MET inhibitors with desirable BBB-penetrating effect to further validate our results.

## Conclusions

Our observations of the changing kinomic landscape and kinase inhibitor responses strongly advocate an overhaul of cancer drug development by considering tumor hypoxia during preclinical settings. Using network analysis, unweighted scoring algorithm, and transcriptomes of clinical tumor specimens, we further enhance the predictive potential of the kinase inhibitor screen to assist in prioritizing potential candidates. Our results encourage drug repurposing of c-MET and PI3K/Akt inhibitors to improve GBM therapy. While GPCs were used in the present study, it is also noteworthy that tumor hypoxia occurs ubiquitously in many solid tumors, entailing the generalizability of our results to other cancers.

## Supplementary Material

Supplementary file legends, figures and tables.

Supplementary file 1.

Supplementary file 2.

Supplementary file 3.

## Abbreviations

CGGA: Chinese Glioma Genome Atlas
GBM: glioblastoma
GPC: glioma-propagating cells
GSC: glioma-stem-like cells
HGF: hepatocyte growth factor
HIF: hypoxia-inducible factor
IPA: Ingenuity Pathway Analysis
NNI: National Neuroscience Institute Singapore
PRECOG: Prediction of Clinical Outcomes from Genomic Profiles
ROS: reactive oxygen species
TCGA: The Cancer Genome Atlas Program
TME: tumor microenvironment
TMZ: temozolomide
