# Supplementary material for: Kinomic profile in patient-derived glioma cells during hypoxia reveals c-MET-PI3K dependency for adaptation
Source: Theranostics. 2021 Mar 5;11(11):5127–42. doi: 10.7150/thno.54741 (PMC8039937; doi:10.7150/thno.54741)
Supplement: Supplementary file 1 — Supplementary file legends, figures and tables. [file thnov11p5127s1.pdf]

Kinomic profile in patient-derived glioma cells during hypoxia reveals c-MET-PI3K dependency for adaptation

Hong Sheng CHENG[1,2], Charlie MARVALIM[1], Pengcheng ZHU[1], Cheng Lui Daniel LAW[1], Zhi Yan Jeremy LOW[1], Yuk Kien CHONG[3], Beng Ti ANG[4,5,6], Carol TANG[1,3,5], Nguan Soon TAN[1,2]

[1]School of Biological Sciences, Nanyang Technological University Singapore, Singapore 637551, Singapore.

[2]Lee Kong Chian School of Medicine, Nanyang Technological University Singapore, Singapore 308232, Singapore.

[3]Neuro-Oncology Research Laboratory, Department of Research, National Neuroscience Institute, Singapore 308433, Singapore.

[4]Department of Neurosurgery, National Neuroscience Institute, Singapore 308433, Singapore.

[5]Duke-National University of Singapore Medical School, Singapore 169857, Singapore.

[6]Department of Physiology, Yong Loo Lin School of Medicine, National University of Singapore, Singapore 117593, Singapore.

Running title: c-MET manipulates antioxidant mechanism in hypoxia.

*** Correspondence**:

H.S.C. Email: hscheng@ntu.edu.sg;

N.S.T. (senior corresponding author) Email: nstan@ntu.edu.sg;

Tel: (65) 6904 1295; Fax: (65) 6339 2889.

Supplemental File

Supplemental File 1. The details of small-molecule inhibitors used in kinase inhibitor screen.

Supplemental File 2: GSEA results of gene expression between peri-necrotic zone and cellular tumor of Ivy GBM Atlas Project RNA-seq data.

Supplemental File 3: GSEA results of gene expression between GBM neoplastic cells over-expressing c-MET and those with low c-MET expression based on single-cell transcriptomes of clinical GBM tumors.

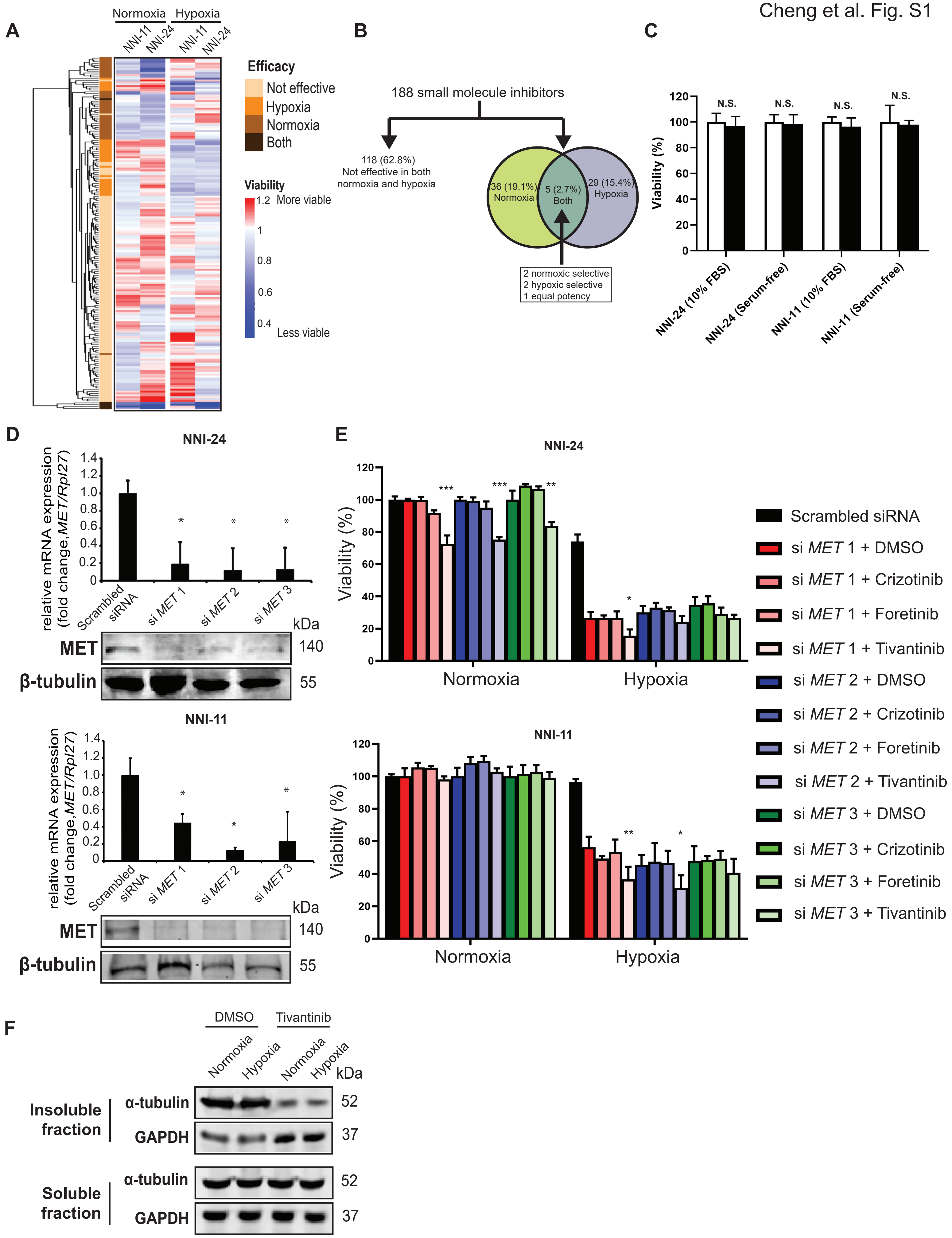
A
Normoxia
Hypoxia
NNI-11
NNI-24
NNI-11
NNI-24
Efficacy
Not effective
Hypoxia
Normoxia
Both
Viability
1.2 More viable
1
0.8
0.6
0.4 Less viable
B
188 small molecule inhibitors
118 (62.8%)
Not effective in both normoxia and hypoxia
36 (19.1%) Normoxia
5 (2.7%) Both
29 (15.4%) Hypoxia
2 normoxic selective
2 hypoxic selective
1 equal potency
C
Viability (%)
N.S.
NNI-24 (10% FBS)
NNI-24 (Serum-free)
NNI-11 (10% FBS)
NNI-11 (Serum-free)
D
NNI-24
relative mRNA expression (fold change,MET/Rpl27)
Scrambled siRNA
si MET 1
si MET 2
si MET 3
kDa
MET
140
β-tubulin
55
NNI-11
E
NNI-24
Viability (%)
Normoxia
Hypoxia
NNI-11
Scrambled siRNA
si MET 1 + DMSO
si MET 1 + Crizotinib
si MET 1 + Foretinib
si MET 1 + Tivantinib
si MET 2 + DMSO
si MET 2 + Crizotinib
si MET 2 + Foretinib
si MET 2 + Tivantinib
si MET 3 + DMSO
si MET 3 + Crizotinib
si MET 3 + Foretinib
si MET 3 + Tivantinib
F
DMSO
Tivantinib
Normoxia
Hypoxia
Normoxia
Hypoxia
kDa
Insoluble fraction
α-tubulin
52
GAPDH
37
Soluble fraction
α-tubulin
52
GAPDH
37

Supplemental Figure Legends

Figure S1. Kinome landscape in hypoxia and off-target analysis of c-MET inhibitors

(A) Heatmap shows the full kinase inhibitor screen data of 188 small molecule inhibitors in GPCs exposed to normoxia and hypoxia. Blue color indicates reduced viability while red color indicates increased viability compared to DMSO-treated cells. The efficacy of each inhibitor in different oxygen environments is shown and color-coded.

(B) Venn diagram summarizes the number and proportion of kinase inhibitors as well as their efficacy in different oxygen environments.

(C) Cell viability assays of NNI-24 and NNI-11 maintained in different culture conditions and exposed to normoxic and hypoxic environments for 72 h. Data are represented as mean±SD from n = 3 replicates. N.S. denotes non-significant.

(D) Relative mRNA (top panel) and protein (bottom panel) expression of *MET* in NNI-24 and NNI-11, whose c-MET was suppressed by three different siRNAs and exposed to normoxic and hypoxic conditions. Data are represented as mean±SD from n = 3 replicates. * $p < 0.05$, ** $p < 0.01$, *** $p < 0.001$.

(E) Viability assays of *c-MET*-knockdown GPCs treated with indicated c-MET inhibitors or vehicle (DMSO) in normoxia and hypoxia. Data are represented as mean±SD from n = 3 replicates. * $p < 0.05$, ** $p < 0.01$, *** $p < 0.001$ compared to cognate DMSO-treated GPCs.

(F) Representative immunoblots of α-tubulin from soluble and insoluble fractions of cell lysates from NNI-24 treated with tivantinib (1000 nM) for 24 h in normoxia and hypoxia. GAPDH which serves as a loading control is from the same samples.

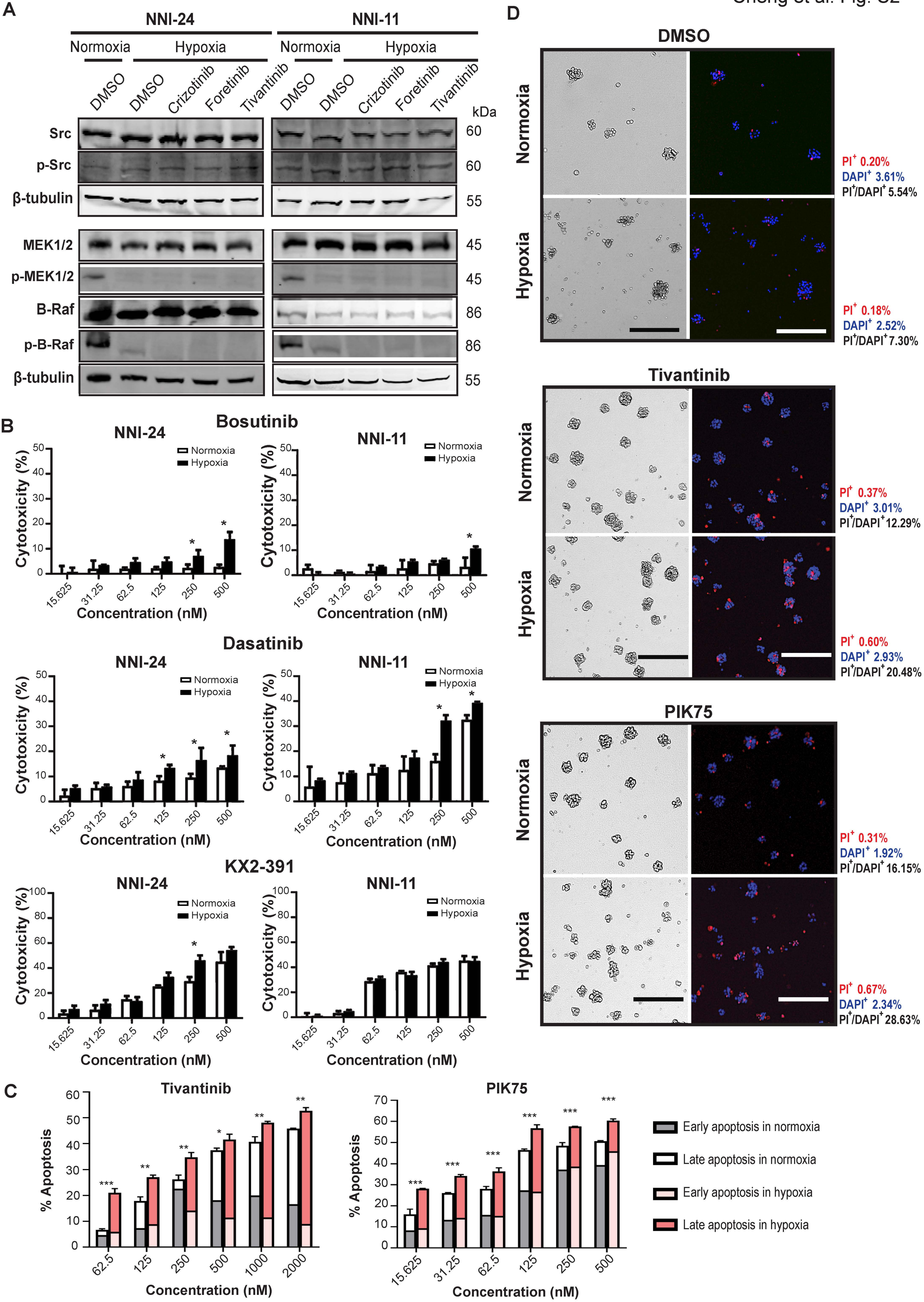
A
NNI-24
NNI-11
Normoxia
Hypoxia
DMSO
Crizotinib
Foretinib
Tivantinib
kDa
Src
p-Src
β-tubulin
MEK1/2
p-MEK1/2
B-Raf
p-B-Raf
60
55
45
86
B
Bosutinib
Dasatinib
KX2-391
Cytotoxicity (%)
Concentration (nM)
15.625
31.25
62.5
125
250
500
C
Tivantinib
PIK75
% Apoptosis
1000
2000
Early apoptosis in normoxia
Late apoptosis in normoxia
Early apoptosis in hypoxia
Late apoptosis in hypoxia
D
DMSO
PI+ 0.20%
DAPI+ 3.61%
PI+/DAPI+ 5.54%
PI+ 0.18%
DAPI+ 2.52%
PI+/DAPI+ 7.30%
Tivantinib
PI+ 0.37%
DAPI+ 3.01%
PI+/DAPI+ 12.29%
PI+ 0.60%
DAPI+ 2.93%
PI+/DAPI+ 20.48%
PIK75
PI+ 0.31%
DAPI+ 1.92%
PI+/DAPI+ 16.15%
PI+ 0.67%
DAPI+ 2.34%
PI+/DAPI+ 28.63%

Figure S2. Analysis of signaling pathways downstream to c-MET in hypoxia.

(A) Immunoblots of indicated proteins from NNI-24 and NNI-11 treated with crizotinib (500 nM), foretinib (500 nM) or tivantinib (1000 nM) and exposed to hypoxia. β-tubulin which serves as a loading control is from the same samples.

(B) Cell viability assays of NNI-24 and NNI-11 treated with increasing concentrations of Src inhibitors and exposed to normoxic and hypoxic conditions. Data are represented as mean±SD from n = 3 replicates. * $p < 0.05$, ** $p < 0.01$, *** $p < 0.001$ compared to corresponding drug concentration in normoxia.

(C) Graphs show percentage of early and late apoptotic NNI-31 (GPC cultured in serum-free environment) induced by tivantinib (c-MET inhibitor) and PIK75 (PI3K inhibitor). Data are represented as mean±SD from n = 3 replicates. * $p < 0.05$, ** $p < 0.01$, *** $p < 0.001$.

(D) Representative brightfield and live/dead fluorescence images of NNI-31 GPCs treated as described in (C). Blue and red indicate live and dead cells, respectively. Percentage of dead (PI^+), live ($DAPI^+$) cells and the ratio of dead/live ($PI^+/DAPI^+$) cells are shown. The scale bar represents 100 µm.

Intracellular ROS analysis of NNI-24

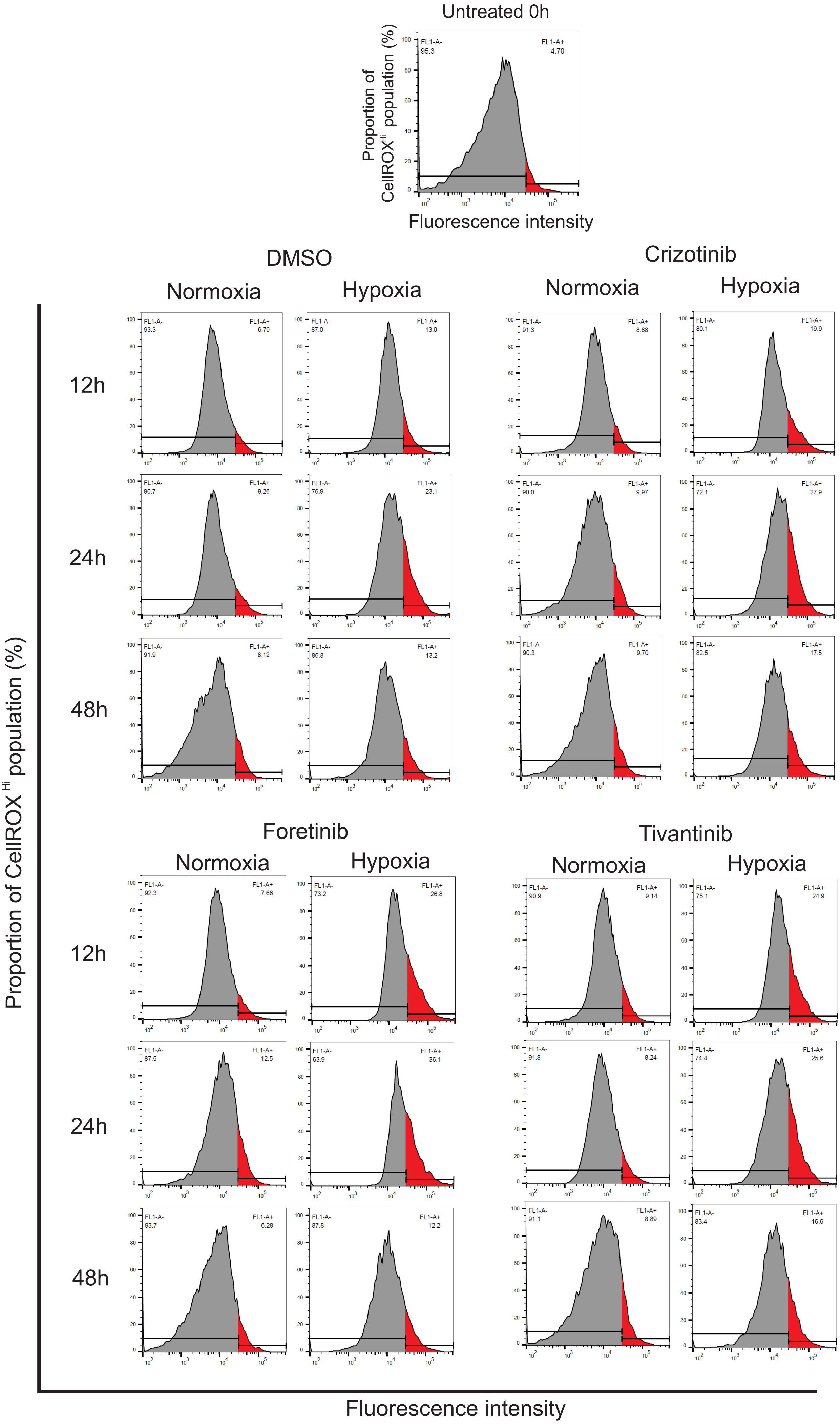

Figure S3. Intracellular ROS analysis of NNI-24.

Histograms show the percentage of NNI-24 cell population with high intracellular ROS ($CellROX^{Hi}$, red area under the curve) when treated with crizotinib, foretinib, and tivantinib for 0, 12, 24, and 48h in normoxia and hypoxia.

Intracellular ROS analysis of NNI-11

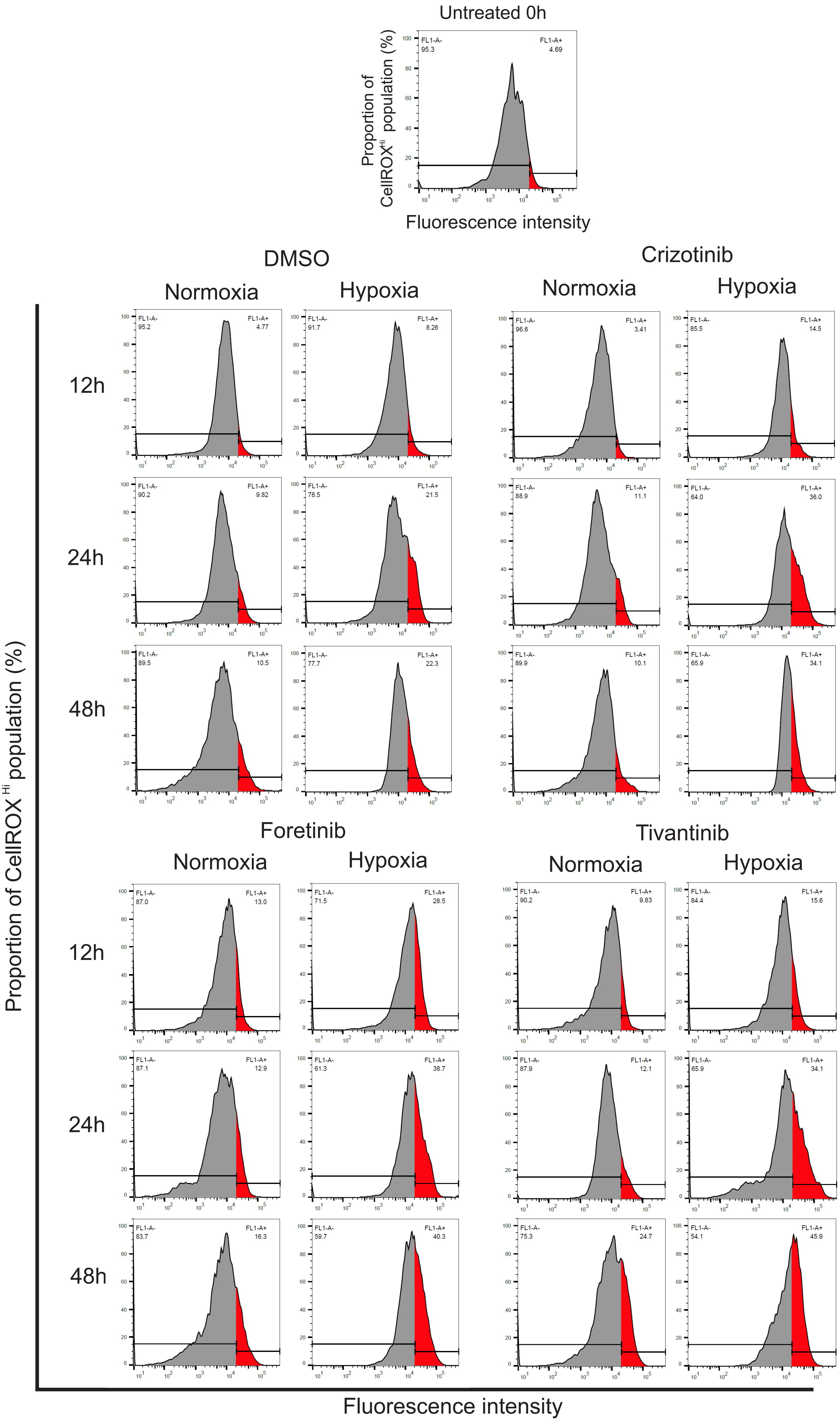

Figure S4. Intracellular ROS analysis of NNI-11.

Histograms show the percentage of NNI-11 cell population with high intracellular ROS (CellROX^{Hi}; red are under the curve) when treated as described in legend of Figure S3.

Cheng et al. Fig. S5

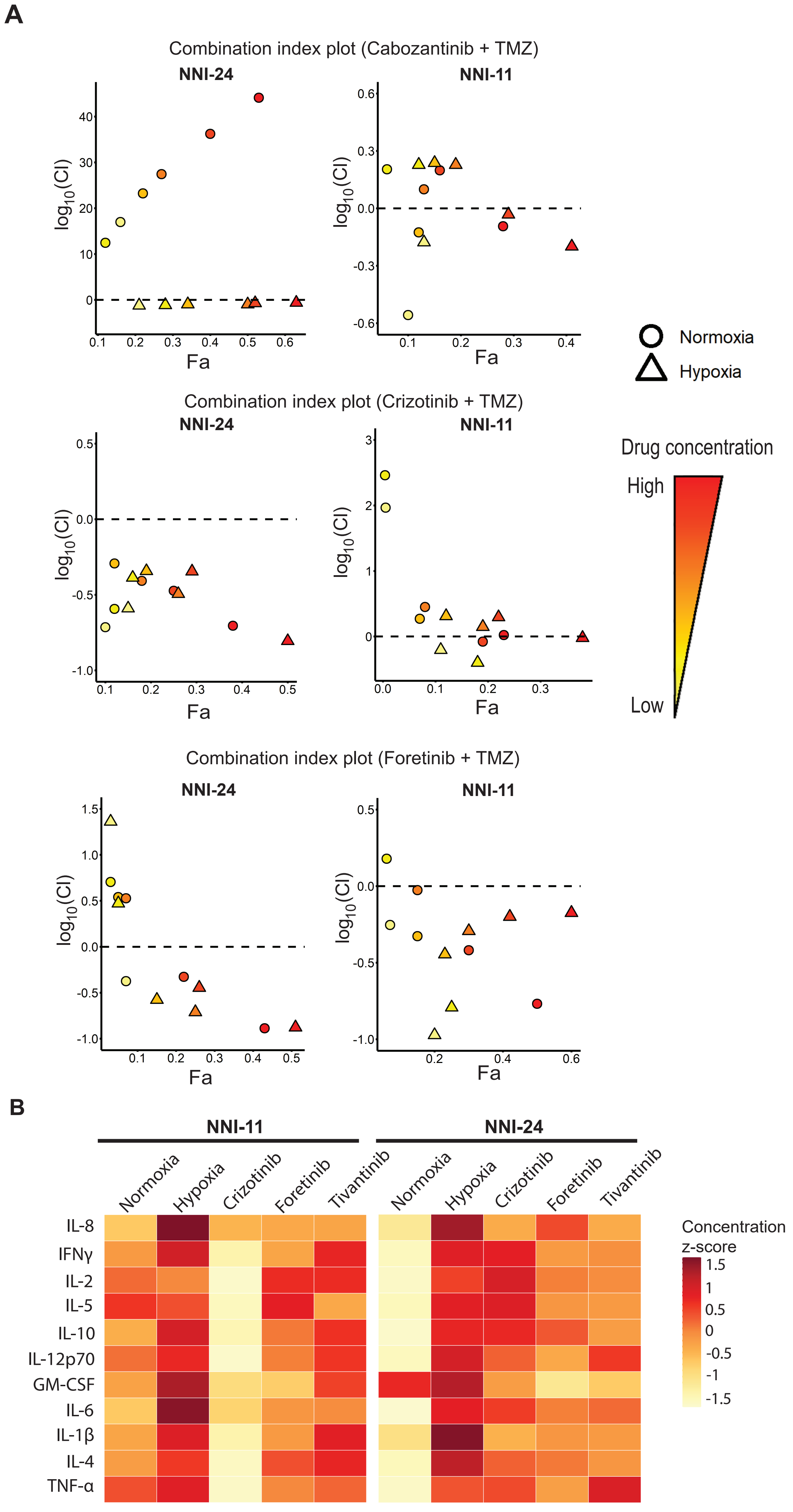

Figure S5. Drug synergism of MET inhibitors and TMZ and cytokine secretion profiling.

(A) Fa-log_{10}(Cl) plots of NNI-24 and NNI-11 treated with combined therapy of cabozantinib, crizotinib, or foretinib with TMZ at increasing concentrations and exposed to normoxic and hypoxic conditions. Drug combinations with $log_{10}(Cl) < 0$ (dotted line) indicate synergism. Increasing color intensity corresponds to increasing concentrations of the drugs.

(B) Heatmaps show cytokine profiles of in culture media from NNI-11 (left panel) and NNI-24 (right panel). The cells (n = 3 replicates) were treated with crizotinib, foretinib and tivantinib and exposed to hypoxia. Vehicle-treated (DMSO) cells exposed to normoxia and hypoxia serve as the control groups. The cytokine concentrations are color-coded and expressed in concentration z-score.

Supplemental Tables

Table S1. The profiling information of GPCs.

GPCs	IDH1 (Direct sequencing)	IDH2 (Direct sequencing)	MCMT promoter (Methylation-specific PCR)	Subtype
NNI-11	Wild-type	Wild-type	Methylated	Proneural
NNI-24	Wild-type	Wild-type	Unmethylated	Classical
NNI-31	Wild-type	Wild-type	Methylated	Proneural

Table S2: qPCR primer sequences

Target genes	Accession number	Forward primer (5'to 3')	Reverse primer (5'to 3')
HGF	NM000601	TCC CTT CTC GAG ACT TGA AAG	CTG CAT TTC TCA TTT CCC ATT
MET	NM001127500	TGA AGT AAT GCT AAA ATG CTG G	CCT ATG GCA AGG AGC AAA GA
NQO1	NM000903	GAA GAG CAC TGA TCG TAC TGG C	GGA TAC TGA AAG TTC GCA GGG
SOD2	NM000636	GCT CCG GTT TTG GGG TAT CTG	GCG TTG ATG TGA GGT TCC AG
PRDX1	NM002574	CCA CGG AGA TCA TTG CTT TCA	AGG TGT ATT GAC CCA TGC TAG AT
GPX1	NM000581	CAG TCG GTG TAT GCC TTC TCG	GAG GGA CGC CAC ATT CTC G
GPX4	NM002085	GAG GCA AGA CCG AAG TAA ACT AC	CCG AAC TGG TTA CAC GGG AA
